# Variation in health beliefs across different types of cervical screening non-participants

**DOI:** 10.1016/j.ypmed.2018.03.014

**Published:** 2018-06

**Authors:** Laura A.V. Marlow, Rebecca A. Ferrer, Amanda J. Chorley, Jessica B. Haddrell, Jo Waller

**Affiliations:** a Cancer Communication and Screening Group, Department of Behavioural Science and Health, UCL, Gower Street, London WC1E 6BT, United Kingdom; b Basic Biobehavioral and Psychological Sciences Branch, Behavioral Research Program, Division of Cancer Control and Population Sciences, National Cancer Institute, Rockville, MD, United States

**Keywords:** Cervical screening, Intention, Uptake, Interventions, Psychological, Beliefs, Attitudes, Abstainer, Unaware, Theory

## Abstract

Understanding factors associated with different types of cancer screening non-participation will help with the development of more targeted approaches for improving informed uptake. This study explored patterns of general health beliefs and behaviour, and cancer-specific beliefs across different types of cervical screening non-participants using the Precaution Adoption Process Model (PAPM). A population-representative sample of women in Britain completed a home-based survey in 2016. Women classified as non-participants (*n* = 839) completed additional questions about health beliefs.

Some general health beliefs and behaviours, as well as cancer-specific beliefs, were associated with particular types of non-participation. For example, those who scored higher on fatalism were more likely to be unaware of screening (*OR* = 1.74, *95%CI*: 1.45–2.08) or unengaged with screening (*OR* = 1.57, *CI*: 1.11–2.21). Women with greater deliberative risk perceptions were less likely to be unengaged with screening (*OR* = 0.74 *CI*: 02.55–0.99) and less likely to have decided against screening (*OR* = 0.71, *CI*: 0.59–0.86). Women who had seen a general practitioner in the last 12 months were less likely to be unaware (*OR* = 0.49, *CI*: 0.35–0.69), and those reporting cancer information avoidance were more likely to be unengaged with screening (*OR* = 2.25, *CI*: 1.15–4.39). Not wanting to know whether one has cancer was the only factor associated with all types of non-participation.

Interventions to raise awareness of screening should include messages that address fatalistic and negative beliefs about cancer. Interventions for women who have decided not to be screened could usefully include messages to ensure the risk of cervical cancer and the relevance and benefits of screening are well communicated.

## Highlights

• Lower risk perceptions are associated with being unengaged with screening or deciding not to attend.
• Fatalistic beliefs are associated with being unaware or unengaged with screening.
• Avoiding cancer information is associated with being unengaged with screening.
• “Not wanting to know” is associated with all types of non-participation.

## 1 Introduction

Cancer screening involves testing for higher risk of asymptomatic early stage cancer or precancerous lesions, which can then be diagnosed and treated before cancer develops. Population-based screening for colorectal, breast, and cervical cancer, along with oral cancer screening for at-risk groups, is recommended by the World Health Organization (WHO, 2013). Key to the success of all cancer screening is participation of the asymptomatic, healthy individual, but uptake of cancer screening is considered sub-optimal across different cancers and different delivery systems (NHS Digital, 2016; von Wagner et al., 2011; White et al., 2017).

Interventions to improve overall uptake of cancer screening have had limited success (Everett et al., 2011; Wardle et al., 2016) and support has been growing for a move from ‘one-size-fits-all’ interventions to more tailored or targeted approaches (Kreuter and Wray, 2003; Myers et al., 2007; Sohl and Moyer, 2007). There has also been a shift in focus towards improving informed choice in cancer screening, ensuring that individuals have a good understanding of the risks and benefits before deciding about participation (Entwistle et al., 2008). Historically, most models of health behaviour (Ajzen and Fishbein, 1980; Maddux and Rogers, 1983; Rosenstock, 1966) have suggested a single set of variables could predict whether a person participates in a health behaviour (Weinstein, 1988). While these models seem to do a good job at predicting how those who are aware of a health threat form a decision about a related behaviour, they offer less insight into the processes involved for those who are unaware of the threat or those who need to translate their intentions into action (Weinstein et al., 2008). Consequently, Weinstein proposed the Precaution Adoption Process Model (PAPM) (Weinstein, 1988) as a way of highlighting different stages of participation (or non-participation) in a health behaviour. The PAPM describes how for any ‘hazard’ there will be several ‘stages’ through which people move before participating in a behaviour to reduce that hazard. They may be unaware (stage 1: unaware), following which they may remain unengaged (stage 2: unengaged). There may then be a period that includes being undecided about whether to participate (stage 3: undecided), before forming an intention or plan (stage 5: decided to act), and then translating this into behaviour (stage 6: acting). A decision not to act can also be made (stage 4: decided not to act). For ongoing behaviours there is also a stage relating to whether the behaviour is being continued (stage 7: maintained). Weinstein proposed that there are qualitative differences between people at different stages and suggested that understanding the variables relevant to each stage could contribute to the design of more effective interventions. Initially developed to explain radon testing behaviour, the PAPM has since been applied to a range of behaviours including osteoporosis prevention and smoking cessation (reviewed here (Weinstein et al., 2008)), and more recently to cancer screening (Costanza et al., 2005; Ferrer et al., 2011; Hester et al., 2015; Marlow et al., 2017).

The PAPM is well suited to cancer screening behaviour because it draws together a range of empirical findings, including the fact that many people who are eligible for screening are unaware or unengaged (Robb et al., 2010) and that there is a significant gap between intention to be screened and participation in screening (Sheeran, 2002). It also allows a dedicated space for those who have made a choice not to participate and therefore works well with the move towards encouraging informed choice in the context of cancer screening (Entwistle et al., 2008). While the term ‘stage’ is used to highlight the phases people move through, the PAPM differs from earlier stage models (e.g. Prochaska and Velicer, 1997) by accepting that there is no set duration for each ‘stage’ and that people may skip stages or may move back into earlier stages. These assumptions work well within the cancer screening context, where the behaviour is repeated every few years, and movement between ‘stages’ before, after and between screening rounds (backwards and forwards) may occur. This provision allows for decisions about participation to change throughout the period over which an individual is eligible for screening. For a more detailed description of how each stage might be applied to cancer screening behaviour see Marlow et al. (2017).

A basic premise of the PAPM is that there are common barriers among people in the same stage and that barriers differ between stages. A number of studies have found support for this in the context of colorectal cancer screening. For example, social cognition variables (perceived risk, worry and regret) are better at explaining intention to be screened, whereas factors relating to life difficulty are better at predicting whether this intention was translated into action (Power et al., 2008). More specifically, those who are unaware or unengaged with colorectal cancer screening are less likely to have seen a health professional recently and have poorer self-rated health (Costanza et al., 2005). Moreover, the unaware are more fatalistic (Costanza et al., 2005), the unengaged are less worried and report lower perceived risk (Costanza et al., 2005; Ferrer et al., 2011), and those who have decided to be screened have higher self-efficacy scores and a greater correlation between risk perceptions and worry (Hester et al., 2015). These studies suggest that the PAPM provides a useful framework for considering different types of non-participant at colorectal screening and understanding differences in health beliefs between types of non-participant. The PAPM has not been applied to cervical screening before and therefore in the current study we further the application of the PAPM to cervical screening and i) explore the pattern of health beliefs across types of non-participant and ii) consider the contribution that different general health beliefs and behaviours, and cancer-specific beliefs, can make in explaining an individual's non-participant type. Since this survey was cross-sectional we have described different ‘types’ of non-participant, rather than referring to stages. We did not form any hypotheses since no studies had explored differences between PAPM stages in the context of cervical screening or in a country outside of the US with free universal healthcare.

## 2 Methods

### 2.1 Participants

In the UK, women aged 25–64 years receive invitations for cervical screening every 3 or 5 years. We commissioned six waves of data collection among screening-eligible women across Great Britain in January/February 2016. Fieldwork was outsourced to a market research agency (TNS) as part of an omnibus survey (i.e. where data are collected during one interview on behalf of multiple clients). Stratified random location sampling was used to select sampling points across Britain. Interviewers knocked on doors at properties in each location, inviting people to take part. Three doors were left between each interview. At each location, quotas were set for employment status and presence of children in the household. Response rates are not recorded by the market research agency. Ethical approval was granted by the UCL Research Ethics Committee (ref: 7585/001).

Data were collected using face-to-face computer-assisted personal interviews (CAPI). A series of four questions assessed awareness of screening, past screening behaviour and future intention to be screened (Box 1). Women were classified into one of six stages of participation (based on Weinstein (Weinstein, 1988)). A detailed explanation of these questions and a flow-diagram indicating how women were allocated to each PAPM stage is available elsewhere (see Fig. 2 of Marlow et al., 2017). The present article focuses on differences in health perceptions among women who were classified as cervical screening non-participants. Women who were up-to-date with screening and intended to re-attend were not asked questions about their health perceptions and were excluded from these analyses. The decision not to include questions about heath beliefs for all women was cost-based. Findings relating to socio-demographic differences between the non-participant types have been published elsewhere (Marlow et al., 2017).

Box 1

1. ‘In the UK, women who are aged between 25 and 64 are invited to participate in the NHS cervical screening program’ followed by ‘Have you ever heard of cervical screening, also known as the smear test or Pap test?’ This was accompanied by a photograph of a woman being screened
  (Response options: yes/no/don't know)
2. [Those who responded yes to Q1] ‘Have you ever had a cervical screening test?’
  (Response options: yes/know/don't know)
3. [Those who responded yes to Q2] ‘When was the last time you had a cervical screening test?
  (Response options: within the last 3 years/3–5 years ago/longer than 5 years ago/don't know).
4. [Those who responded yes to Q1] ‘Do you intend to go when next invited?’
  (Response options: yes/no/don't know + ‘I've never thought about it’ as an additional response for those who had not been screened before)

Alt-text: Box 1

### 2.2 Measures

Women who were classed as cervical screening non-participants were asked questions assessing their general health beliefs and behaviours and cancer-specific beliefs. Items were selected to cover a range of aspects previously shown to be associated with general health outcomes and cancer screening behaviours (see Supplementary Table 1). Items assessing general health beliefs and behaviours included: GP attendance, self-rated health, trust in the doctor, following of medical advice, perceived body awareness, general fatalism (i.e. belief that life events are predestined and beyond the individual's control (Straughan and Seow, 1998; Powe and Finnie, 2003)), future orientation (the extent to which one thinks about or considers the future (Strathman et al., 1994)) and information seeking behaviour. Items assessing cancer-specific beliefs included: knowledge of cervical cancer risk factors, family history (of cervical cancer), perceived risk, cancer fatalism (i.e. belief that getting cancer is beyond the individual's control (Powe and Finnie, 2003)), belief that cancer is a death sentence, not wanting to know about having cancer and cancer information avoidance. Women who had heard of and engaged with cervical screening were also asked about their knowledge and beliefs about cervical screening. As we were interested in understanding women's existing beliefs, those who had never heard of or thought about cervical screening (unaware/unengaged women) were not asked these questions.

#### 2.2.1 Socio-demographic characteristics

Age and marital status were assessed using items designed by TNS or based on the 2011 census. Social grade represented the occupation of the Chief Income Earner in the household: AB managerial/professional; C1 supervisory; C2 skilled manual; D semi-skilled/unskilled manual; E casual/lowest grade workers (Ipsos, 2009). Ethnicity was assessed using the question from the 2011 census (ONS, 2011).

### 2.3 Analysis

Women were excluded if they reported having had a hysterectomy or cervical cancer (*n* = 369), were over 60 years old and living in Scotland (*n* = 27; cervical screening stops at 60 years in Scotland) or provided insufficient data to determine their screening status (*n* = 152). ANOVA and chi-squares were used to explore overall mean/proportion differences across non-participant types. Where overall differences were present, logistic regression compared each of the non-participant types with those who had formed an intention to be screened (intenders; reference group). Odds Ratios (ORs) and 95% Confidence Intervals (CI) are reported. Since most variables are continuous, the OR represents the change in odds of being a particular non-participant type for each point on the scale (Hosmer et al., 2013).

Multivariate models were run for each non-participant group (relative to the intenders), using a step-wise approach including socio-demographics, followed by psychological variables (significant in univariate analyses). This gave an overall estimate of how much these variables contributed to explaining each non-participant type. For non-intenders a third step was included adding attitudes to screening.

## 3 Results

Of the 3113 women who completed the survey, 855 women were classified as screening non-participants. A small number were undecided about screening (*n* = 16) and were excluded, leaving 839 for further analyses. These women were: unaware of screening (*n* = 254), unengaged with screening (*n* = 41), had decided not to be screened (*n* = 118) or were intending to be screened but currently overdue (*n* = 426). Sample characteristics are reported in Table 1. Overall there were significant differences across the four types of non-participant for each of the health beliefs, with the exception of cancer knowledge (Table 2).

**Table 1:** Sample characteristics of non-participants (*n* = 839).

	*n*	%
Age		
25–34	336	40.0
35–44	235	28.0
45–54	153	18.2
55–64	115	13.7
Social grade		
AB	113	13.5
C1	218	26.0
C2	160	19.1
D	190	22.6
E	158	18.8
Ethnicity		
White British/Irish	503	60.0
Any other White	107	12.8
South Asian	121	14.4
Black	70	8.3
Mixed/other ethnicity	32	3.8
Non-participant type		
Unaware	254	30.3
Unengaged	41	4.9
Decided not to be screened^a^	118	14.1
Intending to be screened	426	50.8

a Including *n* = 34 women who were currently up to date but had decided not to attend when next invited.

**Table 2:** Differences in health beliefs between types of cervical screening non-participants.

	Unaware (*n* = 254)	Unengaged (*n* = 41)	Decided not to be screened (*n* = 118)	Intending to be screened (*n* = 426)	F, χ^2^ or t (*p*-Value)
	Mean (SD) or % (*n*)	Mean (SD) or % (*n*)	Mean (SD) or % (*n*)	Mean (SD) or % (*n*)	
General health beliefs and behaviours					
Seen GP in last 12 months (% yes)	61.2 (142)	63.4 (26)	73.0 (84)	76.3 (318)	17.86 (<0.001)
Self-rated health (% good or excellent)	73.9 (156)	80.0 (32)	60.6 (66)	73.0 (300)	8.91 (0.030)
Trust in doctor (range: 1–5)	3.93 (0.77)	3.70 (0.99)	3.55 (1.05)	3.83 (0.76)	5.47 (0.001)
Follow medical advice (range: 1–5)	3.86 (0.82)	3.77 (0.99)	3.44 (1.14)	3.79 (0.95)	5.07 (0.002)
Body awareness (range: 1–5)	3.77 (0.86)	3.65 (1.08)	3.76 (0.81)	3.47 (0.96)	6.41 (<0.001)
General fatalism (range: 1–5)	3.33 (0.93)	3.24 (1.10)	2.77 (1.08)	2.79 (1.00)	15.55 (<0.001)
Future orientation (range: 1–5)	3.82 (0.73)	3.47 (1.06)	3.34 (0.94)	3.76 (0.76)	11.15 (<0.001)
Information seeking (range: 1–5)	3.76 (0.78)	3.23 (1.21)	3.52 (1.07)	3.70 (0.89)	5.02 (0.002)
Cancer-specific beliefs					
Knowledge of cervical cancer risk factors (range: 0–8)	3.29 (2.86)	3.32 (2.60)	3.36 (2.38)	3.54 (2.40)	0.54 (0.654)
Cervical cancer in family (% yes)	5.3 (13)	7.3 (3)	9.6 (11)	18.1 (77)	42.00 (0.001)
Deliberative risk (range: 1–7)	3.37 (1.49)	2.94 (1.64)	2.98 (1.56)	3.57 (1.30)	5.08 (0.002)
Experiential risk (range: 1–5)	2.84 (1.18)	2.13 (0.99)	2.26 (1.17)	2.77 (1.06)	9.34 (<0.001)
Affective risk (range: 1–5)	1.84 (1.13)	1.29 (0.68)	1.52 (0.97)	1.95 (1.00)	10.92 (<0.001)
Cancer fatalism (range: 1–5)	3.14 (1.04)	3.29 (1.04)	2.92 (1.08)	2.84 (1.08)	4.72 (0.003)
Cancer is a death sentence (range: 1–5)	3.10 (1.13)	2.80 (1.11)	2.69 (1.16)	2.82 (1.05)	4.18 (0.006)
I would not want to know if I had cancer (range: 1–5)	2.75 (1.18)	2.73 (1.26)	2.62 (1.39)	2.09 (1.15)	16.85 (<0.001)
Cancer information avoidance (% yes)	18.5 (42)	39.0 (16)	26.3 (30)	22.2 (92)	9.45 (0.024)
Cervical screening beliefs					
Benefits of cervical screening (range 1–5)	–	–	3.72 (0.71)	4.24 (0.57)	8.01 (<0.001)
Screening is embarrassing (range 1–5)	–	–	3.47 (1.29)	2.89 (1.18)	4.45 (<0.001)
Screening is painful (range 1–5)	–	–	3.27 (1.14)	2.70 (1.04)	4.89 (<0.001)
Purpose of screening (range 1–5)	–	–	2.45 (1.22)	2.22 (1.07)	1.89 (0.060)
Cervical screening norms (range 1–11)	–	–	5.86 (2.10)	6.05 (2.04)	0.83 (0.408)

Data collected in Great Britain in 2016.

### 3.1 Unaware

In the multinomial logistic regression with those intending to be screened as the reference group, three of the general health belief and behaviour items were associated with being unaware of screening (Table 3). Women who had seen a GP in the 12 last months were less likely to be unaware of screening (*OR* = 0.49, *CI*: 0.35–0.69). Those who scored higher on body awareness were more likely to be unaware of screening (*OR* = 1.43, *CI*: 1.19–1.73), as were those with higher general fatalism (*OR* = 1.74, *CI*: 1.45–2.08). There were also associations with several cancer-specific beliefs. Those with family experience of cervical cancer were less likely to be unaware of screening (*OR* = 0.25, *CI*: 0.14–0.46). Higher cancer fatalism was associated with greater odds of being unaware (*OR* = 1.31, *CI*: 1.11–1.55) and associations were in the same direction for believing cancer is a death sentence (*OR* = 1.26, *CI*: 1.08–1.47) and not wanting to know if one had cancer (*OR* = 1.56, *CI*: 1.35–1.80). Alongside socio-demographics, general health beliefs and behaviour explained around 27% of the variance in being unaware of screening and this increased to 30% when the cancer-specific beliefs were added to the model (Table 4).

**Table 3:** Predictors of being each non-participant type (univariate ORs and 95% CIs).

	Unaware v intending to be screened	Unengaged v intending to be screened	Decided not to be screened v intending to be screened
General health beliefs and behaviours			
Seen GP in last 12 months (B)	0.49 (0.35–0.69)	0.54 (0.27–1.06)	0.84 (0.53–1.35)
Good self-rated health (B)	1.05 (0.72–1.53)	1.48 (0.66–3.31)	0.57 (0.37–0.88)
Trust in doctor	1.16 (0.94–1.44)	0.83 (0.57–1.21)	0.69 (0.55–0.87)
Follow medical advice	1.09 (0.90–1.30)	0.98 (0.69–1.39)	0.71 (0.58–0.87)
Body awareness	1.43 (1.19–1.73)	1.24 (0.87–1.77)	1.42 (1.12–1.80)
General fatalism	1.74 (1.45–2.08)	1.57 (1.11–2.21)	0.98 (0.80–1.21)
Future orientation	1.10 (0.88–1.38)	0.65 (0.45–0.96)	0.56 (0.44–0.71)
Information seeking	1.08 (0.90–1.31)	0.61 (0.44–0.84)	0.81 (0.65–1.02)
Cancer-specific beliefs			
Cervical cancer in family (B)	0.25 (0.14–0.46)	0.36 (0.11–1.19)	0.48 (0.25–0.93)
Deliberative risk	0.90 (0.79–1.03)	0.73 (0.57–0.93)	0.74 (0.64–0.87)
Experiential risk	1.06 (0.91–1.24)	0.58 (0.42–0.79)	0.66 (0.54–0.80)
Affective risk	0.91 (0.77–1.07)	0.39 (0.23–0.66)	0.62 (0.48–0.79)
Cancer fatalism	1.31 (1.11–1.55)	1.49 (1.09–2.04)	1.08 (0.88–13.2)
Cancer is a death sentence	1.26 (1.08–1.47)	0.98 (0.73–1.32)	0.89 (0.73–1.08)
I would not want to know if I had cancer	1.56 (1.35–1.80)	1.53 (1.19–1.98)	1.44 (1.21–1.70)
Cancer information avoidance (B)	0.80 (0.53–1.20)	2.25 (1.15–4.39)	1.25 (0.78–2.02)

Data collected in Great Britain in 2016.

OR = Odds Ratio, CI = confidence interval

Note: since most variables are continuous the OR represents the change in odds of being in the group for each point on the scale (predominantly from 1 to 5). B indicates that the variable was binary and the OR represents the odds of being in this group.

**Table 4:** Predictors of being unaware, unengaged and deciding not to get screened.

	Unaware	Unengaged	Decided not to be screened
Model 1: socio-demographics			
N	676	466	541
Model X^2^(df)	117.35 (11)	25.05 (11)	78.85 (11)
R^2^ (Nagelkerke)	0.21	0.12	0.21
Model 2: general health beliefs^a^			
N	569	412	481
Step X^2^(df)	17.73 (8)	20.66 (8)	28.69 (8)
Model X^2^(df)	122.38 (19)	42.80 (19)	100.92 (19)
R^2^ (Nagelkerke)	0.27	0.23	0.29
Model 3: cancer-specific beliefs^a^			
N	486	389	448
Step X^2^(df)	26.15 (7)	44.23 (7)	37.54 (7)
Model X^2^(df)	99.43 (18)	72.95 (18)	102.35 (18)
R^2^ (Nagelkerke)	0.27	0.38	0.32
Model 4: general health and cancer-specific beliefs^a^			
N	458	362	423
Step X^2^(df)	33.04 (15)	48.58 (15)	52.92 (15)
Model X^2^ (df)	106.51 (26)	71.28 (26)	116.46 (26)
R^2^ (Nagelkerke)	0.30	0.43	0.37
Model 5: general health, cancer-specific and cervical screening beliefs^b^			
N	–	–	409
Step X^2^(df)			40.71 (3)
Model X^2^(df)	–	–	159.16 (29)
R^2^ (Nagelkerke)	–	–	0.50

Data collected in Great Britain in 2016.

Reference group = intending to be screened.

a Socio-demographics (age, social grade and ethnicity) were entered first.

b Socio-demographics (age, social grade and ethnicity), general health and cancer-specific beliefs were entered first.

### 3.2 Unengaged

Higher general fatalism was associated with being unengaged (*OR* = 1.57, *CI*: 1.11–2.21). In addition, higher future orientation and information seeking scores were associated with lower odds of being unengaged (*OR* = 0.65, *CI*: 0.45–0.96 and *OR* = 0.61, *CI*: 0.44–0.84 respectively). Among the cancer-specific items, greater perceived risk was associated with being less likely to be unengaged (Deliberative: *OR* = 0.74, *CI*: 02.55–0.99; Experiential: *OR* = 0.58, *CI*: 0.42–0.79; Affective: *OR* = 0.39, *CI*: 0.23–0.66). In addition, higher scores on cancer fatalism and not wanting to know if one had cancer were associated with higher odds of being unengaged (*OR* = 1.49, *CI*:1.09–2.04 and *OR* = 1.53, *CI*:1.19–1.98 respectively). Women who reported avoiding cancer information were more likely to be unengaged (*OR* = 2.25, *CI*: 1.15–4.39). Alongside socio-demographics, general health beliefs and behaviour explained around 23% of the variance in being unengaged with screening and this increased to 43% when the cancer-specific beliefs were added to the model (Table 4).

### 3.3 Deciding not to be screened

Reporting good/excellent health was associated with lower odds of deciding not to be screened (*OR* = 0.57, *CI*: 0.37–0.88). Women who reported greater trust in the doctor and usually following medical advice were less likely to have decided not to be screened (*OR* = 0.69, *CI*: 0.55–0.87 and *OR* = 0.71, *CI*: 0.58–0.87 respectively) and those with higher perceived body awareness were more likely to have decided not to be screened (*OR* = 1.42, *CI*: 1.12–1.70). Those with greater perceived risk were less likely to have decided against screening (Deliberative: *OR* = 0.71, *CI*: 0.59–0.86; Experiential: *OR* = 0.66, *CI*: 0.54–0.80; Affective: *OR* = 0.62, CI: 0.48–0.79). Not wanting to know if one had cancer was associated with higher odds of deciding not to be screened (*OR* = 1.44, *CI*: 1.21–1.70). Socio-demographics and general health beliefs and behaviour explained around 29% of the variance in deciding not to be screened and this increased to 37% when the cancer-specific beliefs were added to the model (Table 4).

Higher perceived benefits of cervical screening were associated with lower likelihood of deciding not to be screened (*OR* = 0.27, *CI*: 0.19–0.40) and higher perceived barriers were associated with greater likelihood of deciding against screening (*OR* = 1.50, *CI*: 1.25–1.80 and *OR* *=* 1.64, *CI*: 1.33–2.02 for embarrassment and pain respectively). Adding these items as an additional step in the multivariate model increased the variance explained to 50%.

## 4 Discussion

This study applied the PAPM to cervical screening and identified unique psychological characteristics of different types of screening non-participant, including those who were unaware and unengaged with screening as well as those who had made a decision either to be screened or not to be screened. This is the first study to explore differences in health beliefs across PAPM types for cervical screening. Moreover, it is the first time the PAPM has been applied to cancer screening outside of the US, in a context where screening is offered as part of an organized programme. We found key differences between non-participant types suggesting ways in which the content and delivery of interventions to increase cervical screening uptake may need to vary across different types. The findings presented here complement our paper exploring socio-demographic differences between non-participant types (using the same dataset) (Marlow et al., 2017).

Those who were unaware of screening were the most fatalistic about cancer prevention and had the most negative attitudes towards cancer, consistent with previous studies in the context of colorectal cancer screening (Costanza et al., 2005; Ferrer et al., 2011). The unengaged women were slightly less fatalistic, but still more so than those intending to be screened. The cross-sectional nature of the survey means we cannot determine causality; while it may be that these women have low awareness of screening so do not think there is anything that can be done to avoid cancer, it is also possible that fatalistic beliefs drive avoidance of health information and contribute to low awareness of screening. The associations with general fatalism suggest these beliefs may preclude awareness, at least to some extent. Highlighting the availability of screening and communicating positive messages around cancer prevention may help to reduce fatalism in this group. Since the current written materials do not seem to be reaching these women and they are less likely to have seen a GP recently, using alternative channels (e.g. television or radio campaigns) could be beneficial. One study showed a video intervention designed to decrease fatalism and increase knowledge of colorectal cancer was effective (Powe and Weinrich, 1999). Another avenue to consider might be a community-based empowerment intervention which would educate, motivate and enable women, ensuring they have the means and the motivation to access screening services (Hou and Cao, 2017).

Different psychological variables seemed to play a role for women who had decided not to be screened. Fatalistic beliefs were *not* associated with deciding against screening. Instead these women had lower cancer risk perceptions, suggesting they did not feel screening was relevant to them. In addition, we found that these women were less likely to trust a doctor or to follow medical advice, perhaps suggesting negative previous experiences with the healthcare system. Ensuring risk perceptions are accurate may be important to ensure that women who have decided not to be screened have made an informed decision. Addressing negative health perceptions about doctors and medical advice more generally could also be important for facilitating screening among these women. Future work might explore these previous negative experiences. This might lead to improvements in the service for all women and fewer women deciding not to re-attend.

The unengaged women were similar to the unaware women in some respects, but similar to those who had decided not to be screened in others. Higher fatalistic beliefs and lower perceived risk were associated with being unengaged. As might be expected, the unengaged women were less likely to report seeking information about health and more likely to avoid cancer in the media. This active avoidance means they will likely be a difficult group to access. Given the relatively small size of the unengaged type, it is unlikely they will be the primary target for interventions, but rather they may be “mopped up” by campaigns aimed at those who are unaware and those who have decided not to participate.

Age, social grade and ethnicity explained around 20% of the variance for unaware women and those who had decided not to be screened. This suggests that factors associated with economic conditions and life stage play an important role in understanding some of the different types of screening non-participants. Along with socio-demographics, general health beliefs and behaviour explained a similar proportion of the variance for women who were unaware, unengaged and those who had decided not to be screened (23–29%). Cancer-specific beliefs explained a greater proportion of the variance in the unengaged and decided not to be screened groups (38% and 32%) than the unaware group (27%). The overall model for deciding not to be screened, which included cervical screening attitudes, explained 50% of the variance, which is similar to previous studies exploring attitudes as predictors of intention (Armitage and Conner, 2001). The items we included were predominantly informed by previous research and health behaviour theories which have not focused on the unaware as a unique type of non-participant. Future work might explore the role of different variables in earlier PAPM stages, perhaps trait variables such as conscientiousness, or scales assessing life difficulties and access to social support.

A better understanding of the socio-demographic and psychological characteristics of screening non-participants can contribute to more effective tailoring or targeting of interventions (Kreuter and Skinner, 2000). One option is to use a woman's socio-demographic characteristics to predict which non-participant type she is most likely to be and send her intervention materials with appropriate content for that group (‘targeted’ communication). As technology advances, assessing individual ‘stage’ of non-participation is likely to become more feasible, making the tailoring of messages to individual women a more viable option. For example, smart phone apps are now being used by GP practices in the UK and this mechanism could be used to ask direct questions about awareness and behaviour (e.g. using the PAPM algorithm). Women could then be allocated to a particular non-participant group opening up the opportunity for individually tailored messages.

### 4.1 Limitations

There are several imitations to this study. The sample was population-representative but there are likely to be some response biases. In addition, we do not have a response rate and therefore cannot comment on how generalizable the sample is likely to be. We used self-reported screening uptake and intention to assess PAPM stage because we did not have access to medical records. In addition the items assessing health beliefs were not from a previously validated psychometric tool. The survey was also cross-sectional, so we cannot be sure of the causal direction of some of the associations identified.

Women who were intending to be screened were generally less fatalistic and less negative about cancer, scoring above the mean on perceived benefits of cancer screening and below the mean on barriers. We did not ask questions about health beliefs to women who attended screening (a cost-based decision), so not all stages of the PAPM are explored in the survey and while we can comment on differences between some types of non-participant, we were not able to explore differences in health beliefs between those who intend to be screened and those who participate.

The PAPM suggests that analysis should consider the difference between those who are undecided and those who have decided not to participate, in parallel with comparisons between the undecided and the decided to act. These analyses would offer insight into what contributes to progress from being undecided to forming a decision to act or not. Very few women were undecided about cervical screening so we were not able to examine the data in this way, but this suggests that if we can make women aware of screening, many of them will go on to make a decision.

## 5 Conclusion

Weinstein et al. describe how the PAPM offers a “skeleton” that “needs to be fleshed out for each behaviour” (Weinstein et al., 2008). Here we report the first attempt to do this for cervical screening, providing evidence that health beliefs may vary across different types of non-participant, in line with the PAPM. Our findings suggest that developing interventions to raise awareness of screening should include messages that address fatalistic and negative beliefs about cancer. In addition, interventions for women who have decided not to be screened may need to include messages that clearly communicate the risk of cervical cancer and the relevance and benefits of screening.

## Conflict of interest

The authors declare there is no conflict of interest.

## Appendix A Supplementary material

Supplementary material Image 1
