# Supplementary material for: Variation in health beliefs across different types of cervical screening non-participants
Source: Prev Med. 2018 Jun;111:204–9. doi: 10.1016/j.ypmed.2018.03.014 (PMC5945192; doi:10.1016/j.ypmed.2018.03.014)
Supplement: Supplementary file 1 — Supplementary material [file mmc1.docx]

Supplementary Table 1: Details of items used (wording, response options and original source)

|  | Item | Wording and response options | Source |
| --- | --- | --- | --- |
| A | Seen GP in last 12 months | “Have you visited your GP in the last 12 months” (*Yes*/*no*/*I’m not registered with a GP)* | Developed for this survey |
| A | Self-rated health | “In general how would you rate your health?” (5-point scale: *Excellent* to *Terrible)* | Eriksson et al., 2001 |
| A | Trust in doctor | “I trust the advice given to me by my doctor” | Cockburn et al., 1987 |
| A | Follow medical advice | “I usually follow medical advice (e.g. about having vaccinations, going to appointments or taking medication) without necessarily weighing up all the risks and benefits myself” | Developed for this survey |
| A | Body awareness | “I would know if there was anything wrong with my health” | Cockburn et al., 1987 |
| A | General fatalism | Mean of two items: “Everything that happens is part of God's plan” and “If bad things happen it is because they were meant to be” | Lyratzopouet et al., 2015 |
| A | Future orientation | “I’m prepared to make sacrifices now for benefits in the long run” | Strathman et al., 1994 |
| A | Information seeking | “I seek out information on how to stay healthy” | Guvenc et al., 2011 |
| B | Knowledge of cervical cancer risk factors | A total score of 8 items assessing cervical cancer risk factors (possible range 0-8). A score of 1 was allocated for each item where agree or strongly agree was selected. | Simon et al., 2012a |
| B | Cervical Cancer in family | “Have any friends or family members that are close to you ever been diagnosed with cervical cancer?” *(yes*/*no*/*don’t know)* | Developed for this survey |
| B | Deliberative risk | “Compared to other women your age, what do you think are your chances of getting cervical cancer at some point in the future?” (7-point scale: *much below average* to *much above average)* | Weinstein 1987. |
| B | Experiential risk | “I feel very vulnerable to cervical cancer” | Ferrer et al., 2016 |
| B | Affective risk | “How often do you worry about your chance of getting cervical cancer?” (5-pont scale: *never* to *very often*) | Ferrer et al., 2011 |
| B | Cancer fatalism | “There is not much you can do to lower your chances of getting cervical cancer” | Weinstein et al. 2007 |
| B | Cancer is a death sentence | “A diagnosis of cancer is a death sentence” | Simon et al., 2012b. |
| B | I would not want to know if I had cancer | “I would not want to know if I had cancer” | Simon et al., 2012b. |
| B | Cancer information avoidance | “Do you avoid reading stories or watching programmes about cancer?” *(yes*/*no*/*don’t know)* | Vrinten et al., 2017 |
| C | Benefits of screening | Mean of two items: “Attending cervical screening is beneficial to your health” and “Attending cervical screening is important for peace of mind” | Waller et al., 2009 |
| C | Screening is embarrassing | “Cervical screening is embarrassing” | Waller et al., 2009 |
| C | Screening is painful | “Cervical screening is painful” | Waller et al., 2009 |
| C | Purpose of screening | “Cervical screening is for women with symptoms” | Waller et al., 2009 |
| C | Cervical screening norms | “Thinking about all the women in Britain who are invited to have cervical screening, roughly how many do you think take part?” (11-point scale: *none*, *1 in 10* etc. up to *everyone*). | Developed for this survey |

A=General health beliefs and behaviours; B=Cancer specific beliefs; C=Cervical screening beliefs (asked to those aware of cervical screening only)

Unless otherwise indicated items were responded to on a 5-point scale (strongly disagree to strongly agree)

**References**

Cockburn,J., Fahey,P., & Sanson-Fisher,R.W. (1987). Construction and validation of a questionnaire to measure the health beliefs of general practice patients. *Fam.Pract.*, *4*(2), 108-116.

Eriksson,I., Unden,A.L., & Elofsson,S. (2001). Self-rated health. Comparisons between three different measures. Results from a population study. *Int.J Epidemiol.*, *30*(2), 326-333.

Ferrer,R.A., Hall,K.L., Portnoy,D.B., Ling,B.S., Han,P.K., & Klein,W.M. (2011). Relationships among health perceptions vary depending on stage of readiness for colorectal cancer screening. *Health Psychol.*, *30*(5), 525-535.

Ferrer,R.A., Klein,W.M., Persoskie,A., Avishai-Yitshak,A., & Sheeran,P. (2016). The Tripartite Model of Risk Perception (TRIRISK): Distinguishing Deliberative, Affective, and Experiential Components of Perceived Risk. *Ann.Behav.Med*, *50*(5), 653-663.

Guvenc,G., Akyuz,A., & Acikel,C.H. (2011). Health Belief Model Scale for Cervical Cancer and Pap Smear Test: psychometric testing. *J Adv.Nurs.*, *67*(2), 428-437.

Lyratzopoulos,G., Liu,M.P., Abel,G.A., Wardle,J., & Keating,N.L. (2015). The Association between Fatalistic Beliefs and Late Stage at Diagnosis of Lung and Colorectal Cancer. *Cancer Epidemiol.Biomarkers Prev.*, *24*(4), 720-726.

Simon,A.E., Wardle,J., Grimmett,C., Power,E., Corker,E., Menon,U., Matheson,L., & Waller,J. (2012a). Ovarian and cervical cancer awareness: development of two validated measurement tools. *J Fam.Plann.Reprod.Health Care*, *38*(3), 167-174.

Simon,A.E., Forbes,L.J., Boniface,D., Warburton,F., Brain,K.E., Dessaix,A., Donnelly,M., Haynes,K., Hvidberg,L., Lagerlund,M., Petermann,L., Tishelman,C., Vedsted,P., Vigmostad,M.N., Wardle,J., & Ramirez,A.J. (2012b). An international measure of awareness and beliefs about cancer: development and testing of the ABC. *BMJ Open.*, *2*(6).

Strathman, A., Gleicher, F., Boninger, D. S., & Edwards, C. S. (1994). The consideration of future consequences: Weighing immediate and distant outcomes of behavior. *Journal of Personality and Social Psychology, 66,* 742–752.

Vrinten,C., Boniface,D., Lo,S.H., Kobayashi,L.C., von,W.C., & Waller,J. (2017). Does psychosocial stress exacerbate avoidant responses to cancer information in those who are afraid of cancer? A population-based survey among older adults in England. *Psychol.Health* 1-13.

Waller,J., Bartoszek,M., Marlow,L., & Wardle,J. (2009). Barriers to cervical cancer screening attendance in England: a population-based survey. *J Med Screen.*, *16*(4), 199-204.

Weinstein,N.D. (1987). Unrealistic optimism about susceptibility to health problems: conclusions from a community-wide sample. *J Behav.Med*, *10*(5), 481-500.

Weinstein,N.D., Kwitel,A., McCaul,K.D., Magnan,R.E., Gerrard,M., & Gibbons,F.X. (2007). Risk perceptions: assessment and relationship to influenza vaccination. *Health Psychol.*, *26*(2), 146-151.
